# Unfulfilled psychosocial needs of the adolescent siblings of patients with cancer and the identification of the related factors

**DOI:** 10.3389/fpsyg.2022.983980

**Published:** 2022-09-21

**Authors:** Zeynab Masoudifar, Maryam Rassouli, Hadis Ashrafizadeh, Ensieh Fathollah Zadeh, Nasrin Dadashi, Leila Khanali Mojen

**Affiliations:** ^1^Pediatric Congenital Hematologic Disorders Research Center, Research Institute for Children’s Health, Shahid Beheshti University of Medical Sciences, Tehran, Iran; ^2^Cancer Research Center, Shahid Beheshti University of Medical Sciences, Tehran, Iran; ^3^Student Research Committee, Faculty of Nursing, Dezful University of Medical Sciences, Dezful, Iran; ^4^College of Nursing and Health Sciences, Flinders University, Bedford Park, SA, Australia; ^5^Department of Nursing, Pediatric Congenital Hematologic Disorders Research Center, Research Institute for Children’s Health, Shahid Beheshti University of Medical Sciences, Tehran, Iran

**Keywords:** cancer, psychosocial needs, sibling, Iran, nursing oncology, psychology

## Abstract

**Background:**

The diagnosis of cancer in a child is a stressful experience for the entire family, particularly for adolescent or young adult siblings and their psychosocial needs (PSNs) may remain unfulfilled. The aim of the study was to assess the unfulfilled PSNs of the adolescent siblings of patients with cancer in Iran and examine the relationships between demographic and medical variables and PSNs.

**Methods:**

This descriptive analytical study was conducted in 2019 in seven teaching hospitals in Tehran, Iran. Participants were 188 adolescent siblings of patients with cancer. Sampling was performed consecutively. Data were collected using a demographic and clinical characteristics questionnaire and the Sibling Cancer Needs Instrument (SCNI). To investigate the relationship between demographic variables and the mean score of PSNs subscales, first, the correlation was determined, and afterward, the significant variables were analyzed using multiple linear regression.

**Results:**

In total, 180 adolescent siblings completed the study. Their mean age was 15.66 ± 2.55 years and the mean summary score of their PSNs was 121.15 ± 32.73. Around 80.60% of adolescents indicated at least one unmet need related to each question. The most common unfulfilled needs of participants were related to the information about sibling’s cancer dimension (INFO) (mean: 2.94 ± 0.79) and the less common unfulfilled needs were related to the practical assistance dimension (UFAM) (mean: 2.38 ± 0.93). Based on the regression coefficients, a significant relationship was observed between the mean summary score of PSNs with the duration of cancer (β = –2.199, *p* = 0.006) and mother’s age (β = –2.805, *p* = 0.029).

**Conclusion:**

The adolescent siblings of patients with cancer have different unfulfilled PSNs, particularly respecting information about their siblings’ cancer and support for emotional coping. Family members and healthcare providers should provide these adolescents with strong informational support and fulfill their needs in order to promote their health and their emotional coping.

## Introduction

As a chronic debilitating disease, cancer is the third leading cause of death in adults and the second leading cause of death in children younger than 14 years (World Health Organisation [WHO], 2018). Among children ages 0–14 years, it is estimated that in 2021, 10,500 will be diagnosed with cancer and 1,190 will die of the disease. Among adolescent’s ages 15–19 years, about 5,090 will be diagnosed with cancer and about 590 will die of the disease. The most common types of cancer in children (ages 0–14 years) are leukemia, followed by brain and other CNS tumors, lymphomas, neuroblastoma, kidney tumors, and malignant bone tumors (Miller et al., 2021). Since 1975, in US the overall incidence of cancer for unknown reasons in pediatric and adolescent cancer age ranges has increased by 0.7% per year (Siegel et al., 2020). The incidence rate of cancer among children and adolescents in Iran has also increased from 8.81 to 10.4% in males and from 11.29 to 13.4% in females (Shabani et al., 2020).

Family is the most important source of emotional and social support for patients with cancer and family members have significant roles in managing cancer-related problems (Levesque, 2016; Johansen et al., 2018). A diagnosis of cancer in a child or adolescent results in many challenges and necessitates a shift in roles and responsibilities for all family members (Peek and Melnyk, 2010; Grant et al., 2012; Ward et al., 2014; Dambi et al., 2015; Erker et al., 2018; Khademi et al., 2019; Neugebauer and Mastergeorge, 2021). In addition, they may suffer from psychological problems such as depression, anxiety, anger, fear, concern (Khanjari et al., 2012; Hydary, 2015). Frequent hospitalizations, long-term caregiving, and inability to effectively manage cancer-related problems due to the lack of financial and social support impose heavy caregiver burden on family members (Levesque, 2016; Johansen et al., 2018; Nemati et al., 2018).

Compared with younger siblings, adolescent siblings may be particularly impacted by these changes resulting in physical, financial, social strains (Ward et al., 2014). The reduced attention they receive from parents and extended family members may result in these adolescent siblings feeling jealous and angry, and needing to compete with their ill sibling (Prchal and Landolt, 2012; McDonald et al., 2015; Yang et al., 2016; Cheung et al., 2020; Wawrzynski et al., 2021). These siblings often complain of their parents’ lack of time for them and often experience negative feelings and impaired quality of life (Cheung et al., 2020; Pariseau et al., 2020; Wawrzynski et al., 2021).

Better understanding of the family situation coupled with increased responsibilities placed on adolescent siblings renders them more vulnerable and less able to cope emotionally with the changes (McDonald et al., 2015). Previous studies report that these adolescent siblings experience anxiety, sleep and appetite changes, anger, and a sense of guilt (Patterson et al., 2011, 2014; Prchal and Landolt, 2012). The greater focus of family on fulfilling the needs of its cancer-afflicted member causes adolescent siblings senses of isolation and negligence and exposes them to psychosocial distresses 3–6 times more than their healthy peers (Ghofrani et al., 2019). In addition to the challenges associated with a diagnosis of cancer in a sibling, these adolescents may also experience puberty-related problems and changes (McDonald et al., 2015). Which can further exacerbate psychological problems such as imbalance, instability, irritability, despair, anxiety, and depression, and social disorders (Barrera et al., 2002; Clinton-McHarg et al., 2010; Farahani et al., 2016; Torabi et al., 2017). It has been reported that 65% of adolescent siblings of patients with cancer experience mental health issues (Barrera et al., 2002).

Because of the different effects of cancer, the adolescent siblings of patients with cancer have different psychosocial needs (PSNs) which are often neglected. PSNs are the needs for help and support to maintain and improve emotional and mental health. The fulfillment of the PSNs of the siblings of patients with cancer is a component of comprehensive family-centered and palliative care (Australian Government, Cancer Australia, and CanTeen, 2008; Alderfer et al., 2010). Unfulfilled PSNs affect the normal life and roles of adolescents and cause them problems such as extreme fear, discomfort, apprehension, anxiety, anger, fatigue, and despair. The severity of these problems increases over time if PSNs remain unfulfilled (Australian Government, Cancer Australia, and CanTeen, 2008; McDonald et al., 2015; Patterson et al., 2017). Previous studies reported that the most common unfulfilled PSNs of the adolescent siblings of patients with cancer are the need for information about the disease, the need for involvement in the process of care delivery, the need for communication with the patient, and the need for peer support (Alderfer and Hodges, 2010; Patterson et al., 2011; Long et al., 2015). Conducting a needs assessment in adolescent siblings of cancer patients is an important step in revealing their unfulfilled needs (Ghofrani et al., 2019). Since no study has been conducted for this purpose in Iran and there is no information about the needs of this group of adolescents. Therefore, the aim of the study was to assess the PSNs of the adolescent siblings of patients with cancer and examine the relationships between demographic and medical variables and PSNs.

## Materials and methods

### Design and participants

This descriptive analytical study was conducted in 2019. The setting of the study was seven hospitals affiliated with Tehran University of Medical Sciences, Tehran, Iran, which provided outpatient and inpatient care services to patients with cancer.

In total, 188 eligible adolescent siblings were recruited through consecutive sampling. Inclusion criteria were age 12–19 years, a sibling with a definitive diagnosis of cancer established at least 6 months before the study, awareness of sibling’s cancer diagnosis, and absence of any disorder in physical and mental health based on doctor and family diagnosis and without medical record in this field. Exclusion criteria were fear of cancer stigma in some Iranian subcultures and incomplete response to the study instruments and voluntary withdrawal from the study.

The main question of the research was to estimate the mean summary PSN therefore Two pilot study was used as a sample to calculate the sample size (Masoudifar et al., 2018; Ghofrani et al., 2019). Sample size was calculated with a confidence interval of 0.95, α = .05*z* = 1.96, d = 1, and σ = 6.5 Accordingly, the sample size calculation formula ($n=\left(z_{1-\alpha /2}^{2}\sigma^{2}\right)/d^{2})$ showed that 163 participants were needed. Considering a probable attrition rate of 5%, sample size was increased to 188.

### Instruments

Study instruments included a demographic and clinical characteristics questionnaire and the Sibling Cancer Needs Instrument (SCNI). The demographic and clinical characteristics questionnaire had eighteen items on participants’ age, gender, educational level, birth rank, and occupation, their parents’ age, educational level, and occupation, and their child with cancer’ age, gender, and duration of cancer in sibling ill. SCNI includes 45 items in seven dimensions, namely information about my sibling’s cancer (INFO) (eight items), time out and recreation (TOR) (six items), practical assistance (PRAC) (three items), support from my friends and other young people (SF/OYP) (eight items), dealing with feelings (FEEL) (eight items), understanding from my family (UFAM) (five items), and my relationship with my sibling with cancer (RSIB) (seven items). Items are scaled on a four-point Likert scale as follows: 1: “No need”; 2: “Low need”; 3: “Moderate need”; and 4: “Strong need” (Patterson et al., 2011). Therefore, the total possible scores of the instrument and its aforementioned dimensions are 45–180, INFO (8 items) 8–32, TOR (6 items) 6–24, PRAC (3 items) 3–12, SF/OYP (8 items) 8–32, FEEL (8 items) 8–32, UFAM (5 items) 5–20, and RSIB (7 items) 7–28, respectively. In order to make accurate comparisons among the mean scores of the dimensions, the total mean score of each dimension was divided by the number of its items and thereby, the total possible score of the dimensions was changed into the 1–4 scale. Higher scores show greater unfulfilled PSNs (Patterson et al., 2013, 2014). We used the Persian SCNI which was translated and psychometrically evaluated in a former study (Masoudifar et al., 2018). For this methodological study, the SCNI was translated into Persian using back-translation and revised according to the comments of the developer of the instrument. Then face validity, content validity, construct validity, internal consistency and the stability of the Persian version of the instrument were measure, by examining a population of 180 adolescents having a sibling with cancer in six hospitals in Tehran, Iran. Confirmatory factor analysis approved the construct validity of the instrument and its seven domains. Cronbach’s alpha was measured as 0.97 for the total instrument and 0.80–0.92 for its seven domains and its test-retest correlation coefficient was 0.88 (Masoudifar et al., 2018).

### Data collection

For data collection, within each of the study sites, we identified eligible siblings of children with a confirmed diagnosis of cancer. First, the parents were contacted, then an appointments were made for them to participate in the study, and questions were asked to the eligible siblings in the presence of the parents. Twenty out of 180 participants completed the study instruments at their homes and returned their completed instruments to us. Participants could call the first author of the study over telephone in order to ask their probable questions.

**TABLE 1:** Demographic and clinical characteristics of the participants.

Characteristics		N	%
Sibling’s age (years)	Early (12–14)	70	38.8
	Mid-(15–17)	50	27.7
	Late (18–19)	60	33.3
Sibling’s gender	Girl	98	54.4
	Boy	82	45.6
Sibling’s educational level	Primary	38	21.1
	Junior secondary	52	28.9
	Senior secondary	55	30.6
	University	35	19.4
Sibling’s occupation	Student	150	83.3
	Self-employed	10	5.6
	Worker	4	2.2
	Other	16	8.9
Number of children in family	2–3	129	71.7
	4–5	33	18.3
	≥5	18	10
Birth order in the family	First	101	56.1
	Second	57	31.7
	Last	22	12.2
Father’s age	≥40	55	30.5
	41–50	94	52.2
	51–60	22	12.2
	≥61	9	5
Father’s occupation	Employee	32	17.8
	Self-employed	78	43.3
	Worker	53	29.4
	Retired	8	4.4
	Other	9	5
Father’s educational level	Illiterate	22	12.2
	Primary	41	22.8
	Junior secondary	34	18.9
	Senior secondary	47	26.1
	University	36	20
Mother’s age	≥30	5	2.7
	31–40	113	62.7
	41–50	51	28.3
	≥51	11	6.1
Mother’s occupation	Employee	9	5
	Housewife	166	92.2
	Retired	1	0.6
	Other	4	2.2
Mother’s educational level	Illiterate	24	13.3
	Primary	37	20.6
	Junior secondary	41	22.8
	Senior secondary	50	27.8
	University	28	15.6
Patient’s age	1–10	113	62.7
	11–20	55	30.5
	21–30	12	6.6
Patient’s gender	Girl	81	45
	Boy	99	55
Cancer duration (years)	1	100	55.5
	2	47	26.1
	3	15	8.3
	≥3	18	10
Cancer type	Leukemia	111	61.7
	Lymphoma	15	8.3
	Musculoskeletal	17	9.4
	Neural	13	7.2
	Other	24	13.3

### Statistical analysis

Data were analyzed using the SPSS software (v. 21.0). Data description was done using mean, standard deviation, and frequency measures. To investigate the relationship between demographic variables and the mean score of PSNs subscales, first, the correlation was determined, and afterward, the significant variables were analyzed at the level of less than 0.01 using multiple linear regression. To investigate the relationship between quantitative variables and the mean score of unmet needs of adolescents were used Pearson correlation test and independent *t*-test. To examine the relationship between nominal and rank qualitative variables or the mean score of unmet needs of adolescents, we used *t*-test, ANOVA, KrusKal-Wallis and Mann-Whitney tests according to normality. Finally, the variables that had significant *P*-Value were entered into the regression model.

### Ethical approval

The Ethics Committee of Shahid Beheshti University of Medical Sciences, Tehran, Iran, approved this study with codes (IR.SBMIU.PHNM.1395.472). Participants were provided with information about the study aim and written informed consent was obtained from them or their parents. Data were handled confidentially. Considering this age of informed consent in Iran is 18 years, the presence of adolescents in this study was done with the permission of parents.

## Results

### Sample characteristics

In total, 188 adolescent siblings of patients with cancer were recruited. Eight participants were deleted in the study due to fear cancer stigma in some Iranian subcultures and incomplete response to instruments. 180 participants were included in the final analysis (response rate: 95.74%). The mean of participants’ age was 15.66 ± 2.55 years and most of them were female (54.4%). The duration of cancer in the patient was approximately 1 year in 55.55% of the cases and the most common diagnosis was blood cancer. Table 1 shows participants’ characteristics.

### Participants’ psychosocial needs

The mean summary score of participants’ PSNs was 121.15 ± 32.73 (in the possible range of 45–180). 145 people, equivalent to 80.60% of adolescents indicated at least one unmet need related to each question.

The least frequently unfulfilled needs of adolescents was related to the practical assistance dimension (UFAM) (mean: 2.38 ± 0.93) and the most frequently unfulfilled needs of adolescents were related to the information about my sibling’s cancer dimension (INFO) (mean: 2.94 ± 0.79) and the dealing with feelings dimension (mean: 2.77 ± 0.86). In the information about my sibling’s cancer dimension, 86.7% of participants reported moderate to strong PSNs, while only 58.9% of participants reported unfulfilled PSNs in the practical assistance dimension (Table 2).

**TABLE 2:** The mean scores of PSNs and their dimensions.

Dimensions	Number of items	Mean ± SD	Mean (1–4) based on Likert	Minimum range of possible	Maximum range of possible	At least one unmet need N (%)
Total	45	121.15 ± 32.73	2.69	45	180	145 (80.6)
Information about my sibling’s cancer	8	23.55 ± 6.32	2.94	8	32	156 (86.7)
Dealing with feelings	8	22.12 ± 6.95	2.77	8	32	138 (76.7)
Support from my friends and other young people	8	20.35 ± 7.08	2.54	8	32	124 (68.9)
My relation with my sibling with cancer	7	19.05 ± 6.09	2.72	7	28	138 (76.6)
Time out and recreation	6	16.57 ± 5.10	2.76	6	24	138 (76.7)
Understanding from my family	5	11.93 ± 4.75	2.52	5	20	111 (61.6)
Practical assistance	3	7.56 ± 2.79	2.38	3	12	106 (58.9)

### Relationship between psychosocial needs scores and other factors

Multiple Linear regression analysis using the Enter method was done to determine the relationship between demographic variables and the mean summary score of PSNs. Variables with significant correlation with PSNs in correlation analysis were entered into the regression model (Tables 3, 4). Based on the regression coefficients, a significant relationship was observed between the mean summary score of PSNs with the duration of cancer (β = –2.199, *p* = 0.006) and mother’s age (β = –2.580, *p* = 0.029). This standardized beta coefficient (β) means that for one unit change in the duration of cancer and mother’s age, the mean score of PSNs decreases by –2.199 and –2.805 units, respectively, in other words, the mother’s age has a higher impact factor (Table 5).

**TABLE 3:** Bivariate correlation between the unfulfilled psychosocial needs and demographic and clinical quantitative variable.

Quantitative variable	*R*	*P*-value
Sibling’s age	–0.202	0.006
Father’s age	–0.136	0.068
Mother’s age	–0.229	0.002
Patients’ age	–0.133	0.075
Cancer duration (Years)	–0.203	0.006

**TABLE 4:** Unfulfilled psychosocial needs according to demographic and clinical variables.

Qualitative rank and nominal variables		Mean ± SD	*P*-value	Test statistics
Sibling’s gender	Girl	122.53 ± 31.39	0.482	–1.039*
	Boy	119.51 ± 34.38		
Sibling’s education level	Primary	135.31 ± 33.61	0.022	–2.755**
	Junior secondary	120.50 ± 32.75		
	Senior secondary	116.63 ± 32.02		
	University	113.85 ± 29.33		
Sibling’s occupation	Student	122.10 ± 33.15	0.007	–1.214**
	Self-employee	123.30 ± 28.39		
	Worker	157.25 ± 14.97		
	Other	101.87 ± 24.16		
Number of children in family	2–3	121.66 ± 33.02	0.930	0.281**
	4–5	118.87 ± 32.10		
	>5	121.66 ± 33.40		
Birth order in the family	First child	122.00 ± 33.34	0.642	–0.857**
	Middle	121.98 ± 33.55		
	Last	115.13 ± 28.18		
Patient’s gender	Girl	118.07 ± 31.90	0.279	–0.897*
	Boy	123.67 ± 33.34		
Mother’s occupation	Employee	120.33 ± 33.05	0.223	–0.069**
	Housewife	121.54 ± 32.51		
	Other	125.75 ± 29.76		
Father’s occupation	Employee	125.06 ± 34.22	0.139	0.534**
	Self-employed	119.89 ± 32.61		
	Worker	126.11 ± 31.54		
	Other	100.00 ± 24.10		
Mother’s educational level	Illiterate	115.29 ± 30.78	0.889	–0.291**
	Primary	119.89 ± 35.02		
	Junior secondary	122.90 ± 29.46		
	Senior secondary	122.68 ± 34.04		
	University	122.57 ± 35.03		
Father’s educational level	Illiterate	114.90 ± 33.88	0.815	0.881**
	Primary	121.21 ± 33.67		
	Junior secondary	122.88 ± 28.76		
	Senior secondary	119.36 ± 33.32		
	University	125.61 ± 34.68		
Cancer type	Myeloma	120.51 ± 33.49	0.353	0.862**
	Lymphatic	120.20 ± 29.34		
	Musculoskeletal	116.70 ± 37.47		
	Neural	136.23 ± 31.59		
	Other	119.70 ± 28.36		

Values are expressed as Mean ± SD. *Based on Mann-Whitney test. (Sibling’s gender, Patient’s gender). **Based on KrusKal-Wallis test. (Birth order in the family, Sibling’s Occupation, Number of Children in Family, Mother’s educational level, Father’s educational level, Mother’s occupation Father’s occupation, Sibling’s Education Level, cancer type).

**TABLE 5:** Results of multiple linear regression analysis between mean summary score of unfulfilled psychosocial needs and demographic and clinical variables.

Outcomes	Mean summary unfulfilled psychosocial needs score				
	
Parameter	Unstandardized coefficients		Standardized coefficients	t	P
				
	B	Std.Error	Beta		
**Sibling’s age**	–0.215	1.641	–0.017	–1.31	0.896
**Cancer duration (years)**	–0.458	0.424	–0.173	–2.199	0.006
**Mother’s age**	–0.932	0.163	–0.200	–2.805	0.029
**Sibling’s occupation**	–4.220	2.700	–0.116	–1.563	0.120
**Sibling’s education level**	–4.540	3.888	–0.143	–1.168	0.245

## Discussion

This study assessed the PSNs of the adolescent siblings of patients with cancer. Findings showed that the total mean score of PSNs was 121.15 ± 32.73. Two former studies in Australia reported that the mean score of PSNs among the siblings of patients with cancer was 103.3 ± 35.1 (McDonald et al., 2015) and 103.89 ± 34.88 (Patterson et al., 2017). These findings imply the higher level of unfulfilled PSNs of the adolescent siblings of patients with cancer in Iran. The diagnosis of a family member by cancer imposes heavy responsibilities on parents and reduces their ability to pay attention to all their other children. Former studies in Iran reported the heavy caregiver burden and the high levels of unfulfilled needs of the families of patients with cancer (Valizadeh et al., 2014; Ashrafian et al., 2018; Motlagh et al., 2019), which denote limited attention to the needs of the other members of these families. Adolescents have specific needs and need great parental attention. However, parents who have a child with cancer may be unable to effectively fulfill the PSNs of their other children. Lack of support and holistic family-centered care in Iran may be another reason for the high level of PSNs in the present study. Parents often lack information, which relates to physicians’ busy schedules, cultural hierarchy related to physicians’ knowledge, their interactive roles and relationships inside and outside the home, Lack of communication between treatment staff and parents and lack of palliative care in Iran (Khanali Mojen et al., 2018; Ghofrani et al., 2019; Khademi et al., 2019). Since palliative care is a human and necessary need for children with cancer and is recognized as one of the six main foundations of cancer control all over the world (Daher et al., 2013). This type of care plays an important role in the management of complications caused by the disease and its treatments for the patient, family and official caregivers (Rassouli and Sajjadi, 2014). Palliative care covers a wide range of people’s problems, and the difference in age, cultural context and support systems, and the degree of disease progression can cause the needs related to palliative care to have a wide range of needs (Abrahm, 2012). In Iran, patient care is mostly patient-focused (Rajabpour and Rayyani, 2019), palliative care services are not efficient and coherent, and hence, the needs of the family members of patients with cancer, particularly their siblings, are often neglected (Khanali Mojen et al., 2018).

The greatest unfulfilled PSN of participants in the present study was the need for information about the IR sibling cancer. In line with this finding, several former studies reported the need for information as an important unfulfilled need of the siblings of patients with cancer (McCarthy, 2011; Nolbris and Ahlström, 2014; Patterson et al., 2014, 2017; Zebrack et al., 2014). Limited access to information is a major factor contributing to this finding. Parents and healthcare providers are two main sources of information for the adolescent siblings of patients with cancer (Ghofrani et al., 2019). However, previous studies in Iran have shown that the need for information about child’s disease was the main unfulfilled need of parents (Valizadeh et al., 2014; Ashrafian et al., 2018; Motlagh et al., 2019). Parents who lack information about their child’s disease cannot effectively fulfill the informational needs of their family members. On the other hand, healthcare providers in Iran cannot effectively fulfill the informational needs of the family members of patients with cancer due to their own heavy workload (Zamanzadeh et al., 2014; Mazhariazad et al., 2019).

In addition, mothers with children suffering from chronic diseases in Iran experience more post-traumatic stress due to the structure of their different duties in the context of the family, more communication with the ill child, and as a result of the pressure caused by their daily needs (Nikfarid et al., 2012; Afsari et al., 2020). As a result, she may not pay enough attention to her siblings and their needs. This makes the mother feel guilty. Guilt is a conscious and painful feeling and is experienced if a person believes, she has acted against his values and laws and neglected her other children (Alderfer and Hodges, 2010; Nikfarid et al., 2012). Accordingly, the informational needs of the adolescent siblings of patients often remain unfulfilled despite its great importance and high prevalence (McDonald et al., 2015; Caprino and Riccardi, 2017).

The need for dealing with feelings was another main unfulfilled PSN of the adolescent siblings of patients with cancer in the present study. Participants reported feelings such as guilt, anxiety, fear, and ineffective coping with fear which highlight their great need for support by psychologists, counselors, and social workers. Similarly, former studies reported that adolescents continuously sought support for effectively dealing with their emotions (Nolbris and Hellström, 2005; Wallin et al., 2016). Ineffective coping and limited parental attention are two main factors which prevent the fulfillment of the need for effective dealing with feelings among the adolescent siblings of patients with cancer. These siblings would benefit psychological counseling in order to learn coping strategies. However, to the best of our knowledge, there is no specific supportive program for these siblings and most healthcare services focus on the fulfillment of the needs of the parents of children with cancer. Moreover, the services of social workers are not community-based and are mainly provided in hospital settings to fulfill the needs of patients (Khanali Mojen et al., 2018).

Practical assistance was the lowest unfulfilled PSN in the present study. A former study also reported the same finding (Patterson et al., 2017). The practical assistance dimension of PSNs pertains to the need for support in areas such as the problems of daily life, household activities, transportation, school assignments, and work as well as changes in household due to the illness of a family member and its treatments (Patterson et al., 2011, 2014). The lowest dimensional mean score of this dimension is attributable to the strong structure of families in Iran in which family relationships are strong enough to support family members and help them cope with their problems (Sajadian et al., 2015). In Iran, informal sources of support such as grandparents, aunts, and uncles are strong enough and effective in helping children cope with their conditions and fulfill their needs and thereby, reduce the needs of the adolescent siblings of patients in the area of practical assistance.

Our findings also showed that mother’s age had a significant negative relationship with participants’ PSNs. In other words, participants with older mothers had significantly lower PSNs. This finding may be due to the greater capacity for older mothers to cope with critical family problems. A former study also showed that older parents had greater knowledge and experience and were more competent in caring for their adolescents (Afshary et al., 2016). We also found that duration of disease had significant negative relationship with participants’ PSNs. When the duration of the disease increases, Adolescent’s understanding of the situation in the family and adaptive behavior also increase. Therefore, the level of needs decreases. Of course, it can be said that this finding is related to cultural issues (Rassouli and Sajjadi, 2014; Khanali Mojen et al., 2018; Masoudifar et al., 2018).

It can be said about the differences between mothers and fathers in what they offer to their children: in Iran, Most mothers are forced to change their lifestyle due to meet the demands of their ill child in hospital and at home. Fathers typically continue to work and care for their healthy children at home while their wives are at the hospital. Therefore, the normal routines of fathers may be less disrupted than those of mothers. However, fathers are also affected by a cancer diagnosis and treatment in their child (Wysocki and Gavin, 2004). The results of studies in Iran show that the burden of mothers’ care in physical and care dimensions was more than the care load of fathers. Also, in the emotional dimension, the burden of caring for fathers was reported more (Valizadeh et al., 2014), which may be due to cultural and geographical differences (Bakas et al., 2001). In other words, the role of the mother in providing physical care for children with cancer at home, managing symptoms and doing housework, but the role of the father is focused on doing outdoor work and emotional support of siblings, other children and the mother of the family (Valizadeh et al., 2014).

### Study limitation

This study had some limitations. In Iran, a diagnosis of cancer is associated with social stigmatization and most parents prefer not to talk with their children about this. Consequently, some adolescents in the study setting had limited awareness of their siblings’ cancer and were ineligible for the study. Moreover, some parents did not agree with the participation of their adolescent children in the present study due to the potential negative emotional effects of participation. These limitations prolonged the process of sampling and data collection. This study did not take other measures into consideration that might affect the PNS, such as the psychiatric condition of the participants (e.g., depressive and anxiety symptoms), and the severe risk of sibling illness, and since mental disorders were one of the variables affecting the findings, so we considered the absence of self-reported mental disorders as one of the inclusion criteria, but accurate measurement of these disorders with valid tools was another of our limitations.

## Conclusion

This study shows that the adolescent siblings of patients with cancer have great need for information about their sibling’s cancer and its treatment and need support for effective emotional coping with their new conditions. Providing comprehensive education and information about the cancer diagnosis, treatment, and its effect on families may be useful to adolescent siblings’ It is also important for siblings to be provided with coping strategies in order to address their psychological and emotional status. Providing family, school, and community based interventions may be particularly helpful in supporting adolescents siblings cope with cancer in their family.

It is suggested that for parents with children with cancer and their siblings, planning for family-oriented nursing care that highlighting specifically that educational programs could both meet the informational needs of parents and siblings should be started in order to take an effective step toward empowerment. Also, nurses can make a significant contribution in improving the quality of family and child care by providing correct answers, paying attention to the concerns of parents and siblings, supporting and providing them with some comfort items. It is also necessary to carry out nursing care based on the nursing process, so that in the first step a comprehensive investigation and recognition is done and the knowledge deficit and ignorance of the parents are identified.

## Data availability statement

The original contributions presented in this study are included in the article/supplementary material, further inquiries can be directed to the corresponding author.

## Ethics statement

The studies involving human participants were reviewed and approved by the Ethics Committee of Shahid Beheshti University of Medical Sciences, Tehran, Iran, approved this study with codes (IR.SBMU.PHNM.1395.472). Written informed consent to participate in this study was provided by the participants’ legal guardian/next of kin, or the participants themselves.

## Author contributions

ZM, LK, MR, HA, EF, and ND involved in the study conception and design. LK, MR, ND, and HA contributed to the data collection and analysis. LK, MR, and HA drafted the manuscript. LK, MR, HA, EF, and ND involved in critical revisions for important intellectual content and administrative and technical support and supervised the work. All authors contributed to the article and approved the submitted version.
